# Being in control of Parkinson’s disease: A qualitative study of community-dwelling patients’ coping with changes in care

**DOI:** 10.1080/13814788.2018.1447561

**Published:** 2018-03-23

**Authors:** Annette O. A. Plouvier, Tim C. Olde Hartman, Anne van Litsenburg, Bastiaan R. Bloem, Chris van Weel, Antoine L. M. Lagro-Janssen

**Affiliations:** a Department of Primary and Community Care, Radboud University Medical Center, Nijmegen, The Netherlands;; b Department of Neurology and Parkinson Centre Nijmegen, Donders Institute for Brain, Cognition and Behaviour, Radboud University Medical Center, Nijmegen, The Netherlands;; c Department Health Services Research and Quality, Australian National University, Canberra, Australia

**Keywords:** Neurology (Parkinson’s disease), general practice/family medicine, patient empowerment, qualitative designs and methods (changes in care)

## Abstract

**Background:** Chronically ill patients have to cope with transfers in the level or setting of care. Patients with prevalent disorders such as diabetes mellitus can be supported by their general practitioner (GP) when experiencing such care changes, as the GP already offers them disease-specific care. For community-dwelling patients with low-prevalent diseases such as Parkinson’s disease (PD) – for which disease-specific care is provided by medical specialists – tailoring support to handle care changes requires more insight into patients’ coping.

**Objectives:** To explore PD patients’ coping with care changes.

**Methods:** A qualitative interview study was performed in 2013–2015 with a purposive sample of 16 community-dwelling PD patients in the Netherlands. A research assistant visited patients every month to explore if they had experienced a care change. If so, patients were interviewed face-to-face. An inductive approach to comparative content analysis was used.

**Results:** Patients encountered a variety of care changes such as changes in the level of unpaid care, the purchase of tools, modification of pharmacotherapy or admission to hospital. Being able to anticipate, initiate and independently handle care changes contributes to patients’ sense of control and acceptance of the post-change situation. Patients, who commenced care changes themselves, had more realistic expectations of it.

**Conclusion:** Community-dwelling PD patients seem to be able to cope with the care changes they face. Offering education to facilitate their anticipation and initiation of changes in care and their ability to act independently, can contribute to patients’ wellbeing. GPs can play a role in this.

KEY MESSAGES

- Community-dwelling Parkinson’s disease patients will face care changes.
- They prefer to solve changes independently, which is facilitated by anticipation and initiation, related to realistic expectations of the post-change situation and leads to a feeling of control.
- Support to handle care changes can also be provided by GPs.

## Introduction

Chronically ill patients will face changes in care at some point in the course of their disease. Patients with Parkinson’s disease (PD), for example, suffer from motor symptoms and a wide variety of non-motor symptoms [1]. The fluctuating expression of symptoms and progression of the disease frequently force PD patients to adapt to new impairments and disabilities [1]. Some of these disabilities will necessitate changes in care: transfers of patients between different levels of (non) professional care within the same setting or between different (healthcare) settings [2,3].

Changes in care may include changes in domestic help, the purchase of a tool or modification of pharmacotherapy [3]. So far, however, research has mainly focused on transitional care from one healthcare setting to another or to home [3–7]. Studies among patients with high prevalent chronic conditions such as heart failure, chronic obstructive pulmonary disease (COPD) and cancer show that patients may feel they lack control when discharged from a hospital. Moreover, they may feel insecure, unprepared, inadequately guided and not involved in care-taking decisions at those moments [6,7].

Offering support to prepare patients to handle changes in care can contribute to patients’ well-being [8]. The insight is growing that patients’ healthcare needs to deal with the challenges of chronic disease are defined not only by the disease itself but also – and perhaps even more so – by patients’ ability to cope [9]. General practitioners (GPs) are the most appropriate healthcare providers to offer medical and mental support to patients with prevalent conditions such as COPD and diabetes mellitus (DM) who experience changes in care, as they are already used to provide disease-specific care to these patients and are familiar with patients’ context [10,11].

For community-dwelling patients with a less prevalent disease such as PD, it is not clear who should be offering support in handling care changes, as the neurologist offers disease-specific care, while the GP has more expertise in offering support related to patients’ circumstances [2,14]. Moreover, patients with PD face extra difficulties in handling changes in care. Disease-related factors such as the diminishing ability to take the initiative and to mentally adapt challenge patients in their ability to cope [12,13]. To offer PD patients tailored support, more insight is necessary into the changes in care PD patients’ encounter and into their coping with these changes. In this study we, therefore, ask ourselves how community-dwelling PD patients, who mostly have mild-staged disease, cope with the care changes they face in the course of their disease.

## Methods

### Study design

We performed an exploratory qualitative study using semi-structured interviews with community-dwelling PD patients in the Netherlands. Purposive sampling of patients was chosen to get a broad view of the different ways patients cope with changes in care. Interviews were chosen as data collection method to gain in-depth insight into the different coping strategies.

The research ethics committee of Radboud University Medical Center examined the study protocol and concluded that the study could be carried out in the Netherlands without requiring approval by the accredited regional research ethics committee (11 December 2013). Written informed consent was obtained from all participating patients.

#### Selection of study subjects

This study was part of a larger project on community-dwelling PD patients’ coping and experiences with changes in care [15,16]. Between September 2013 and November 2014, 15 general practices in and around Nijmegen (the Netherlands) were asked to select patients that met the following criteria: a diagnosis of PD (established by a neurologist according to accepted criteria); community-dwelling; capable of handling a video camera with instructions; and no severe memory problems that the GP expected to interfere with retrospective interviewing [15]. A purposive sample of patients – based on age, gender and severity of PD according to Hoehn and Yahr (H&Y) [17] – was approached by their GP.

When a patient agreed to participate, the researcher AP (a medical doctor with experience in qualitative research) gave information that was more detailed and asked the patient for informed consent. Initially, patients were asked to participate for a period of 1 year. After inclusion of eight patients, we reduced the study period to 6 months because patients mentioned that 1 year was rather too long. The point of data saturation determined the final number of included patients. Data were collected between January 2014 and June 2015.

#### Data collection

Supported by an instruction manual, patients made a video once a fortnight [15]. Once a month, a research assistant visited the patient with a two-fold purpose. On the one hand, the visit was used to collect the patients’ videos, allowing the researchers to get a verbal and non-verbal impression of the physical and mental state of the participating patients. On the other hand, the assistant would ask the patients if they had encountered a change in care, naming some exemplifying changes (Table 1) and stimulating the patient to tell more [15].

**Table 1.** Changes in care named as an example during the visits of the research assistant.

Change in care
A change in the extent of domestic help that is provided A change in the extent of personal care help that is provided A domestic adjustment (such as a bracket on the toilet or shower) The purchase of a specific tool (such as a walker or adapted cutlery) A modification of pharmacotherapy The involvement of a healthcare provider (including, for example, a physical therapist or a speech therapist), who was not involved before Consultation of the GP or medical specialist, if not part of routine follow-up Adaptation of working hours or type of work Admission to specialised day care or hospital

If patients had experienced a change in care, they were interviewed face-to-face in their own home by a skilled interviewer (AP), who had no professional relationship with the patients. A brief topic guide based on expert opinion was available with core questions and optional prompts. This guide was tailored using specific individual information from the patients’ videos, which were not analysed in further detail (Table 2). Interviews were recorded and fully transcribed.

**Table 2.** Semi-structured interview topic guide (initial version^a^).

Can you describe the change in care that took place? Can you tell me about your experiences with this change in care? How did you experience it? Can you describe if anything or anyone influenced your experience? And, if so, how did this influence your experience? Can you tell me about your coping with this change in care? How did you cope with this change in care? Why did you cope with it this way? Can you describe your considerations? Can you describe if anything or anyone influenced your coping? And, if so, how did this influence your coping?

a The guide was adapted as the study proceeded; information from the patient’s videos was used to tailor the guide for each interview.

More detailed information on recruitment and data collection can be found in the study protocol [15].

#### Data analysis

ATLAS.ti 7, a computer programme for qualitative data analysis, was used to support coding the interviews. Analysis of the anonymous transcripts started as soon as the first interviews had been transcribed and was an iterative process using an inductive approach to comparative content analysis [18,19]. Two researchers (AP, AVL) independently read all transcripts and applied codes to meaningful words or sentences. Codes were discussed, seeking agreement for their content. New codes arising from these discussions were applied to the transcripts. Codes were grouped into themes, and final themes were agreed upon with the supervisory committee (all authors). Themes were used to adapt the interview topic guide and to progressively focus and explore data in-depth. After analysis of 16 interviews with 12 different patients, no significant new codes emerged. Conduction and analysis of five additional interviews confirmed saturation.

## Results

### Study population

Of the 15 general practices approached, three did not respond to the request for participation and three were not able to select patients who met the inclusion criteria. The remaining nine general practices selected 41 suitable patients, of whom 35 were willing to be approached by the researcher. Nineteen of these patients decided not to participate because of the burden PD gave them, the expected burden of making videos every fortnight or because of other personal circumstances.

Sixteen patients participated in the study: eight during 1 year and eight during half a year. Three patients did not complete the follow-up period: one patient died, one patient withdrew because of the burden of comorbidity and one patient because of difficulties with storytelling to the video camera. The patients’ mean age at the start of the study was 68 years (SD 6.0). Most patients were male (*N* = 11). Most patients had an H&Y stage between H&Y 1 and H&Y 2.5 at the start of the study; only two patients were in H&Y stage 4 (Table 3).

**Table 3.** Characteristics of the participating community-dwelling patients with Parkinson’s disease – including oversight of the experienced changes in care.

Patient Code (A–J)	Sex	Age at start study (years)	Severity of disease at start study: mild–severe (H&Y stage 1–4)	Experienced change(s) in care (each line represents a separate interview and shows the discussed changes in care)	Follow-up period (months)
A	Male	65	1	Modification of PD-related pharmacotherapy Domestic adjustment	12
B	Female	58	1.5	Purchase of a tool Change in unpaid care Domestic adjustment	12
C	Male	59	1.5	Change in unpaid care	12
D	Male	76	2	Modification of PD-related pharmacotherapy (adverse effects)	12
E	Male	63	2	Purchase of a tool Domestic adjustment	12
F	Male	67	1.5	Acute admission to hospital, modification of PD-related pharmacotherapy	12^a^
G	Male	65	1.5	Involvement of a speech therapist Consultation of GP, consultation of neurologist, modification of PD-related pharmacotherapy	12
H	Male	79	4	Acute admission to hospital	12^a^^,^^b^
I	Female	75	1	Consultation of GP, planned admission to hospital Consultation of neurologist, modification of PD-related pharmacotherapy	6
J	Male	73	2	Domestic adjustment, modification of PD-related pharmacotherapy Consultation of neurologist	6
K	Female	70	4	Consultation of GP, consultation of neurologist, modification of PD-related pharmacotherapy	6
L	Male	72	2.5		6^a^
M	Female	70	1		6
N	Female	64	2.5	Modification of PD-related pharmacotherapy	6
O	Male	65	1.5	Consultation of GP, ENT specialist and neurologist, modification of pharmacotherapy (laxative)	6
P	Male	72	2	Consultation of neurologist, consultation of GP, modification of pharmacotherapy (laxative)	6

a Did not finish follow-up.

b Deceased during follow-up.

Two participating patients did not experience any changes in care (Table 3). Thirteen patients and one personal caregiver (replacing the patient who had died during the study period) were interviewed about 34 changes in care. Some patients were interviewed more than once because they experienced changes at different moments. The interview with the caregiver served to get insight into the type(s) of change the deceased patient encountered. A total of 21 interviews were conducted, each taking between 60 and 90 min.

### Encountered changes in care

Patients faced a variety of changes in care such as changes in the level of unpaid care to prepare meals, the purchase of tools such as an adapted cup and the modification of PD-related pharmacotherapy because of hallucinations. Two patients were admitted to hospital acutely, and one patient had a scheduled admission for further investigation (Table 3).

### Patients’ coping with changes in care

We identified three themes related to patients’ coping that influenced their experiences of changes in care and their acceptance of the post-change results: the ability to anticipate; the ability to initiate; and the ability to act independently. These themes will be explored below and will be illustrated with quotations.

### The ability to anticipate (Box 1)

Changes in care that were not foreseen by patients could be overpowering for them. For example, a patient who had been admitted to the hospital acutely realized that admission was inevitable but felt that his wishes had not been addressed. This contributed to his experienced lack of control at the moment of the change (Q1.1).

Box 1 Quotes of community-dwelling PD patients illustrating the theme ‘The ability to anticipate’.

Quote related to the inability to anticipate
• Q1.1 ‘I had the idea that Parkinson has caused my complaints…I told him [the GP] 10 times: “That is what I believe.” …but he persisted and then I had to go to hospital’ (Male, 67 years, H&Y 1.5).
Quote related to the ability to anticipate
• Q1.2 ‘Well, it [encountering a change in care] isn’t difficult. […] I know it is a progressive disease. […] I anticipate’ (Male, 63 years, H&Y 2).

Patients who expected a change to occur were mostly able to anticipate. Sufficient knowledge of the disease facilitated anticipation (Q1.2).

### The ability to initiate (Box 2)

Patients preferred to initiate changes themselves. They, for example, initiated domestic adjustments, the purchase of a tool or the modification of PD-related pharmacotherapy to find a proper balance between the therapeutic and adverse effects of medication. If patients were able to initiate a change themselves, they had realistic expectations of it, which helped them accept the post-change situation, even if their impairments were not remedied (Q2.1, Q2.2, Q2.3).

Box 2 Quotes of community-dwelling PD patients illustrating the theme ‘The ability to initiate’.

Quotes related to the ability to initiate
• Q2.1 ‘Climbing over the edge of the bathtub is getting more and more difficult … so eventually it [renovating the bathroom] will have to be done. […] Then we better do it now, now I can still handle the inconveniences that come with a renovation’ (Male, 65 years, H&Y 1).
• Q2.2 ‘You cut yourself once, then another time … At first you think that things will be all right … but then you realize it is part of the process you’re in and you make a practical decision’ (Male, 63 years, H&Y 2).
• Q2.3 ‘Well, I wanted something [an electric shaver] that would be more comfortable, so I didn’t have to be afraid of cutting myself with a razor’ (Male, 63 years, H&Y 2).
Quotes related to the inability to initiate
• Q2.4 ‘During a planned visit with the Parkinson’s nurse … I told her I was drooling more and more … Then she said “Well, it can’t hurt to visit the speech therapist”. […] I hoped she [the speech therapist] would have a solution, but she just let me swallow water and told me it was all within the norm’ (Male, 65 years, H&Y 1.5).
• Q2.5 ‘Yesterday I took the pills at 10 pm. At 11 pm, my husband switched off the light. When I wanted to turn over in bed, I was barely able to do it. However, I had just taken those pills! I should have been able to do that [turn over] by then, shouldn’t I?’ (Female, 64 years, H&Y 2.5).
• Q2.6 ‘The effects I expected of the modification of my medication failed to happen … for example, fewer dips or a shorter period; that I would experience less trouble due to dips. Actually, that I would just feel better. […] However, looking back at the past few weeks, I’m disappointed about the effect’ (Male, 65 years, H&Y 1).

If a healthcare provider initiated a change in care, for example the consultation of other healthcare providers or modification of PD-related pharmacotherapy when symptoms got worse, patients had unrealistically high expectations of its results. As these expectations were usually not met, patients were disappointed and had difficulties accepting the impairments that remained after the change (Q2.4, Q2.5, Q2.6).

### The ability to act independently (Box 3)

If patients were able to solve their problems independently, they considered themselves to be the manager of the change in care, which led to a sense of control over the change itself and the situation afterward (Q3.1). This was irrespective of the apparent complexity of the change.

Box 3 Quotes of community-dwelling PD patients illustrating the theme ‘The ability to act independently’.

Quote related to the ability to act independently
• Q3.1 ‘Nowadays I take my medication more knowingly, taking into account that if I take my medication now, I’ll have my ‘low’ in two hours’ time. I have the feeling that I am less affected by it because I manage the situation. […] It’s the feeling that you’re the master of your own fate’ (Male, 76 years, H&Y 2).
Quotes related to the inability to act independently
• Q3.2 ‘Sometimes I’m angry that I’m no longer able to do it myself. […] At those moments, I have the feeling I should still be able to do it. Yes, it makes me sad’ (Female, 58 years, H&Y 1.5).
• Q3.3 ‘It’s hard to be dependent. I am not used to needing help. […] I prefer doing things myself. When you need help, it means you depend on other people’ (Male, 65 years, H&Y 1).
• Q3.4 ‘I think that’s a matter of embarrassment again. What will she [my neighbour] think of me? Will she think that I cannot do anything on my own anymore? […] I do not have that with my husband, because he is so familiar. […] When I ask my neighbour, I feel like I’m a whiner’ (Female, 58 years, H&Y 1.5).
• Q3.5. ‘If you can do it yourself, you can do it whenever you want to and in your own tempo. […] Now, I am more or less obligated to wait until he [my son] has time to do it [offer help]’ (Male, 59 years, H&Y 1.5).
Quotes related to acting together
• Q3.6 ’During the consultation with the neurologist we decided together to change the medication dose’ (Female, 70 years, H&Y 4).
• Q3.7 ‘I don’t want to do that [make the decision]. […] In my opinion, when a healthcare provider [neurologist] tries to help you, you should just listen and do what he says’ (Female, 75 years, H&Y 1).

Loss of the ability to act independently would lead to feelings of anger and grief (Q3.2 and Q3.3). Asking for help was difficult, and patients’ experiences differed depending on the person they asked for help (Q3.4, Q3.5).

If the help of a healthcare provider was inevitable, patients emphasized the importance of shared decision-making as this allowed them to be still involved and to maintain control over the change in care (Q3.6). One patient, however, stressed that, if a healthcare provider was involved, she did not want to be the one making the decision (Q3.7).

## Discussion

### Main findings

Community-dwelling PD patients encounter a variety of changes in care such as changes in the level of unpaid care, the purchase of tools, modification of pharmacotherapy or admission to hospital. These changes have an impact on patients’ lives, no matter the apparent complexity of the change. Three themes related to patients’ coping influence their experiences of the changes in care and the situation afterward: the ability to anticipate, the ability to initiate and the ability to act independently. Being able to anticipate, initiate and independently handle a change in care contribute to a sense of control and acceptance of the post-change situation. Patients with mild-staged disease, who succeed to initiate a change in care, have realistic expectations of it. However, when a healthcare provider initiates a change without explicitly discussing what can be expected, patients’ expectations are unrealistically high and unmet.

### Strengths and limitations

To the best of our knowledge, this study is unique in its focus on community-dwelling PD patients’ coping with the moments changes in care occur. The design of the study has unique features. The monthly visits of the research assistant enabled us to respond quickly to changes in care and to interview patients shortly after these turning points in life, thereby limiting the risk of recall bias. In addition, the information gathered from the patients’ videos was used to tailor the interview topic guide, facilitating in-depth questioning in the interviews [19]. Moreover, our study population varied in sex, age, living circumstances and severity of disease. We succeeded to include two patients with H&Y stage 4; this is unique given the level of disability that comes with such a disease-stage. We, therefore, feel that we were able to make a valid contribution to the knowledge of the changes in care community-dwelling PD patient’s encounter and of their coping with these changes.

However, some limitations need to be taken into account. Most of the included patients had a mild-stage disease. The use of technical equipment and the long duration of data collection might have influenced this. The (expected) burden of making videos was a frequently mentioned reason not to participate in the first place, and one participating patient withdrew early for this reason. Patients willing and able to use technical equipment – such as the patients included in this study – might be more able to initiate and independently handle changes in care. It is possible that non-participants would have encountered more complex care changes that were more difficult to anticipate, initiate or handle independently. Our population, finally, came from a single regional setting in the Netherlands with well-developed specialized PD care. This population was not inclined to consult their GP for PD-related questions, as we know from an earlier study [16]. Patients in different areas might have different care experiences.

### Interpretation in relation to existing literature

#### Resilience

Chronically ill patients’ subjective well-being does not have to be affected by new impairments if they develop successful coping strategies to deal with the problems they face in their daily lives [9,20]. Resilience is crucial in this. Huber et al. have already proposed to view health as ‘the ability to adapt and to self-manage’ [20]. Although studies on successful coping with PD emphasize that patients need to accept that they have a chronic disease that will inevitably lead to disabilities and limitations, and that they need to be realistic about their possibilities, yet search for solutions that limit the impact of disabilities and limitations on one’s personal life, symptoms such as apathy and fatigue challenge patients in their resilience [12,13,21–23]. We were, therefore, pleasantly surprised to find out that most patients in our study were able to independently cope with the problems they encountered by making adaptations in their contexts such as a reconstruction of the bathroom or the purchase of an electric shaver. They anticipated and initiated changes in care themselves, not willing to wait until the disease would take over control by forcing the change to happen. This importance of being in control is in line with other literature on coping with chronic diseases and seems to be independent of the type of disease patients suffer from and the type or complexity of change encountered [6,7,24,25].

#### Expectations

Earlier research on PD patients’ most bothersome symptoms and preferred coping strategies showed that patients have high confidence in medication, physical activity and instrumental support [26]. We found that patients’ expectations of changes in care are closely related to their ability to initiate these changes themselves. Patients who, for example, initiated a modification of their PD medication to find a better balance between the therapeutic effects and side effects had more realistic expectations of the change than patients in whose case the neurologist initiated the same change. This might be related to patients’ level of knowledge of the disease and medication: patients who have better understanding of the way their medication works may be more inclined to initiate a modification themselves and may better comprehend its possible effects. Education might be helpful in this.

#### Education

A study of patients with multiple sclerosis (MS), for example, showed that patients have more positive experiences with changes in care if they better know what to expect of their disease and treatment strategies [25]. As this can help patients with an unpredictable disease such as MS, the same might be true for patients suffering from the progressive disease with fluctuating expression that PD is [27]. PD patients expressed the need for education on the disease and treatment strategies before [26,28]. Our study adds to this by showing that education on possible solutions that fit into the patient’s context can help community-dwelling PD patients handle the changes in care that will inevitably occur during their disease. To anticipate, to initiate and to independently handle care changes, will contribute to PD patients’ resilience and their experienced quality of life and health [20,23].

### Implications for clinical practice

Most of the changes in care the community-dwelling PD patients in our study encountered occurred in the patient’s personal context, and seemed to have more to do with patients’ level of functioning and context-related factors than with disease-specific factors. Therefore, offering support to PD patients to handle changes in care does not have to be limited to neurologists, it can also be provided by healthcare providers with insight into the relevant influencing factors, such as the GP that patients have a long-term relationship with. This is in line with the growing insight that patients’ healthcare needs are more defined by their ability to cope than by their disease [9]. We feel that GPs can fulfil the same supportive role to handle care changes for community-dwelling patients suffering from complex (low prevalent) diseases such as PD as for patients with prevailing conditions such as COPD and DM, although the disease-specific care for these patients is organised differently.

Knowing that PD patients’ coping methods influence their experiences and acceptance of the post-change situation, GPs should focus their support on enabling patients to anticipate, initiate and act independently whenever possible. It is essential for GPs to have knowledge of the disease, treatment and possible care changes to be able to explicitly discuss what can realistically be expected of a care change.

## Conclusion

Community-dwelling PD patients, mainly with mild-staged disease, cope with a variety of changes in care. Being able to anticipate and initiate changes in care, and to act independently during these changes, facilitates patients’ sense of control and the acceptance of the new situation. GPs can enable these coping strategies by offering support, thus stimulating their patients’ resilience.
